# An examination of the usefulness of a quantitative appraisal method in nursing human resource management in primary hospital operating rooms: An example of integrated collaborative scheduling

**DOI:** 10.1097/MD.0000000000037938

**Published:** 2024-05-10

**Authors:** Lijun Ding

**Affiliations:** a Anesthesiology and Surgery Department, Yiwu Central Hospital, Jinhua City, Zhejiang Province, China.

**Keywords:** efficiency, integrated collaborative scheduling, primary hospital, satisfaction

## Abstract

In recent years, China medical and health services have made great development. However, the management of nursing human resources in operating room of primary hospitals still faces a series of challenges. In the nursing work of operating room, high-quality nursing human resource management is important for improving the efficiency of operating room and ensuring the safety of patients. From January 2022 to December 2022, comprehensive collaborative scheduling and quantitative scoring evaluation methods were carried out in our hospital, and relevant data were collected. The flexible scheduling combined quantitative scoring performance appraisal system and the traditional scheduling plus average distribution performance appraisal system were statistically analyzed and compared in terms of annual surgical cases, annual overtime hours, annual back work hours, annual compensatory rest hours, and average daily working hours. This study was based on 30 medical staff (27 females and 3 males) in the operating room of a primary hospital. The annual operation volume increased by 387 cases compared with before, and the attitudes of patients to the service attitude and preoperative waiting time were significantly improved, reaching more than 95%. In addition, in the survey of surgeons, it was found that their satisfaction with preoperative preparation and operation time was significantly higher than that of the traditional scheduling method, and reached more than 95%. In the survey of nursing staff, it was found that the satisfaction with the traditional scheduling method was about 80%, and the satisfaction directly reached 100% after the comprehensive collaborative scheduling system. Based on the above survey, the satisfaction of nurses, doctors and patients with the new comprehensive collaborative scheduling system has improved compared with before. After the implementation of the comprehensive collaborative scheduling system, the annual surgical volume has increased significantly, and the average daily working hours of nursing staff have decreased. Comprehensive collaborative scheduling is an effective method of nursing human resource management in operating room, which can effectively improve the work efficiency of nurses and the satisfaction of patients, doctors and nurses. In practice, this method needs to be continuously explored and refined to adapt to different application scenarios and requirements.

## 1. Introduction

Application of flexible scheduling in nursing staff.

In recent years, China medical and health service has made great development. However, the management of nursing human resources in operating room of primary hospitals still faces a series of challenges.^[1–3]^ In the nursing work of operating room, high-quality nursing human resource management is very important for improving the efficiency of operating room and ensuring the safety of patients. Therefore, how to effectively manage the nursing human resources in the operating room has become an urgent problem to be solved. In this context, quantitative evaluation system was introduced into the management of operating room nursing human resources, aiming to quantify the work performance of nursing staff through the establishment of a scientific evaluation index system, so as to evaluate and motivate personnel performance.^[4–7]^ In addition, comprehensive collaborative scheduling as a new scheduling method, through flexible scheduling, reasonable arrangement of staff working time and post rotation, further improve the efficiency and quality of operating room nursing work.^[8]^ However, at present, the effectiveness of quantitative evaluation system in the operating room nursing human resources management of grassroots hospitals is relatively insufficient, and the impact of comprehensive collaborative scheduling on this system also needs to be further discussed. Therefore, this study aims to evaluate the effectiveness of quantitative evaluation system in the operating room nursing human resource management of primary hospitals through empirical analysis, and explore the optimization effect of comprehensive collaborative scheduling on the system, in order to provide scientific basis and feasible suggestions for optimizing the operating room nursing human resource management.

## 2. Materials and methods

### 2.1. Research object

The subjects of this study were 30 operating room nurses in Yiwu Central Hospital, including 3 males and 27 females. Among them, 13 were undergraduates, 14 were junior college students and 3 were secondary school nurses. There are 1 deputy chief nurse, 10 supervisor nurses, 12 nurses and 7 nurses. To develop comprehensive collaborative scheduling and quantitative scoring evaluation methods in our hospital from January 2022 to December 2022.

### 2.2. Research methods

The annual operation cases, annual overtime hours, annual off-work hours, annual compensatory leave hours, per capita working hours per day were analyzed and compared between the flexible scheduling joint quantitative scoring performance assessment system and the traditional scheduling and average distribution performance system.

#### 2.2.1. New and old scheduling methods

The traditional scheduling methods of surgery are generally: day shift 8:00 to 12:00, 14:00 to 17:30, whole shift 8:00 to 15:30, night shift 17:30, 8:00 the next day. Compared with the traditional scheduling method, the flexible scheduling method increases the flexible shift, that is, the shift of surgical nursing staff is rationally arranged according to the operation volume of the next day, and the number of adjustable mobile nursing staff is increased. Flexible scheduling leave calculation: noon and night surgery do not makeup time off, make up overtime pay. If the operation lasts from the first night to the next night 1h, a half-day leave will be given. Those who attend the operation at night will be given half a day off. This calculation method is easy to avoid the trouble of nurses accumulation of leave which is difficult to count.

#### 2.2.2. Specific quantitative assessment indicators

There are 3 categories, one is the comparison of basic indicators, the other is the evaluation and satisfaction of nurses, doctors and patients, and the third is the comparison of specific surgical indicators, including operation volume, operation time, reception time and error rate.

#### 2.2.3. Quantitative scoring performance assessment methods

Hand-washing itinerant nurses is calculated at 0.4 points per hour, and postoperative care is 0.2 points per unit. If the postoperative care needs to be changed, the score is assigned to the replacement. For those dealing with infectious diseases, if they are handled in strict accordance with the disinfection and isolation system and recorded in detail, each hand-washing itinerant nurse will score 0.1 points. There are more operations in the evening, and the overtime workers are scored according to the operation time, and 2 points are added each time. Patients in critical condition during the operation, the implementation of major and middle rescue, and rescue records in the rescue record book, or more intraoperative blood transfusion, up to or more than 3 bags, while the cost of the computer in time, itinerant nurses score 0.3 points, other nurses involved in rescue score 0.1 points. The main class audit, verification, statistical scoring overtime of 0.5 points per day. Due to special reasons, the above work is not completed, and the corresponding score is not accumulated. If there is negligence in the work, 0.1 points will be reduced each time, 2 points will be reduced each time for general errors, and 5 points will be reduced each time for major errors. Time according to actual work, that is, time from the beginning of anesthesia to the time of leaving the operating room after the patient enters the operating room. The scoring method of time counting is 1 point for 1 hour, 0.5 point for 30 minutes and 0.25 point for 15 minutes. 1d Total score was obtained by adding the time of each operation. Equipment nurses and itinerant nurses scored the same; > 4 hours operation, instrument nurse added 1 point; The overtime hours will be doubled, including the overtime of emergency standby shift, and the night shift will be doubled from 1:00 to 5:00. Five points will be deducted for each falsification. Performance pay is then calculated based on the earned points.

### 2.3. Statistical methods

SPSS25.0 statistical software was used to analyze the data. Measurement data were expressed as mean ± standard deviation, and categorical variables were expressed as count (percentage). When the measurement data did not obey the normal distribution, the median (M), the interquartile range (Q), and the rank sum test were used. Descriptive statistical analysis and one-way analysis of variance were used to analyze the relevant indicators of comprehensive collaborative scheduling and quantitative scoring assessment. Chi-square test was used to evaluate the value of comprehensive collaborative scheduling and quantitative scoring in improving the working efficiency of the operating room and the satisfaction of patients, surgeons and nurses. *P* < .05 was considered statistically significant.

## 3. Results

### 3.1. Comparison of basic quantitative indicators

Systematic comparison of the 2 in terms of annual surgical cases, annual overtime hours, annual unpaid hours, annual compensatory hours and average daily working hours showed that the flexible scheduling system significantly increased the number of annual surgical cases in our hospital, and significantly reduced the daily working hours of operating room nurses. The specific results are shown in Table 1.

**Table 1:** Comparison of basic quantitative indicators.

	The number of surgeries per yr	Annual overtime h	Annual unpaid h	Annual compensatory h	Average working h per d
Traditional scheduling	4533	225	0	225	9.33 ± 1.53
Flexible scheduling	4920	476	186	166	7.69 ± 0.74
t	-	-	-	-	2.33
*P*	-	-	-	-	.134

### 3.2. Patient evaluation

We collected the attitude and evaluation of patients on the system, fully understood the experience of the people served, and understood the advantages and disadvantages of the system from the side. The specific results are shown in Table 2.

**Table 2:** Patient satisfaction results analysis table (%).

	Attitude of service	Preoperative waiting time	Manners and manners	Operation of skill
Traditional scheduling	95 (90.5%)	88 (84.0%)	93 (88.6%)	98 (93.3%)
Flexible scheduling	103 (98.1%)	99 (94.3%)	100 (95.2%)	102 (97.1%)
χ²	5.66	5.91	3.14	1.68
*P*	.017	.015	.076	.195

### 3.3. Doctor and nursing evaluation

The application of flexible scheduling combined with quantitative scoring performance appraisal system in the human resource management of nurses in the operating room has abolished the traditional scheduling and performance equal distribution system, which not only makes rational and effective use of human resources, but also improves the work efficiency of nurses. At the same time, it embodies the fair principles of technical risk and responsibility risk are proportional to performance and remuneration, more work, more pay for better work. It can mobilize the initiative and enthusiasm of nurses and improve the quality of nurses’ work. Through the survey, the results analysis of the service attitude and work quality of the operating doctors to the nurses can be obtained, and the specific results are shown in Tables 3 and 4.

**Table 3:** Physician satisfaction results questionnaire (%).

	Attitude of service	Cooperation in surgery	Preparation of items	Preparation for anesthesia	The speed of operation
Traditional scheduling	66 (89.2%)	69 (93.2%)	70 (94.6%)	65 (%)	69 (93.2%)
Flexible scheduling	70 (94.6%)	72 (97.3%)	73 (98.6%)	70 (94.6%)	73 (98.6%)
χ²	1.45	1.35	1.86	2.11	2.78
*P*	.228	.245	.172	.147	.095

**Table 4:** Nurses’ satisfaction results questionnaire (%).

	Number of people	Performance appraisal indicators		Performance appraisal results	
		Satisfaction	Dissatisfy	Satisfaction	Dissatisfy
Traditional scheduling	30 (100%)	25 (83.3%)	5 (16.7%)	24 (80%)	6 (20%)
Flexible scheduling	30 (100%)	30 (100%)	0	30 (100%)	0
χ²	-	5.45		6.67	
*P*	-	.019		.010	

### 3.4. Comparison of relevant indicators in the operating room

After the application of flexible scheduling system and quantitative scoring system, the relevant indicators of surgery in our hospital are significantly improved compared with before. The advantages and disadvantages of the 2 are presented in the table below, and the specific results are shown in Table 5.

**Table 5:** Related indicators in the operating room.

	Operation volume	Rate of error	Duration of surgery	Reception time
Traditional scheduling	4533	75 (1.65%)	5.25 ± 0.78	52 ± 17.3
Flexible scheduling	4920	46 (0.93%)	5.33 ± 0.88	36 ± 9.2
t	-	9.67	−0.068	0.818
*P*	-	.019	.946	.413

## 4. Discussion

### 4.1. Influence of comprehensive collaborative scheduling on working efficiency of operating room nurses

Through the data analysis of this study, it can be found that comprehensive collaborative scheduling has a significant effect on improving the work efficiency of operating room nurses. Compared with previous studies,^[9,10]^ this study adopts a more reasonable method in scheduling, which can make full use of nurses’ professional skills and experience, so as to relieve the workload of operating room nurses and improve work efficiency. First of all, comprehensive collaborative scheduling can better consider the personal characteristics and skill distribution of nurses. Traditional scheduling methods often only consider the number and time arrangement of nurses, but ignore the differences between individual nurses. However, different nurses have different professional skills and experience levels, and assume different responsibilities and tasks in the operating room. Through comprehensive collaborative scheduling, we can allocate nurses to suitable positions according to their strengths and experiences, so that each nurse can play their own advantages and improve work efficiency. Second, comprehensive collaborative scheduling can be more flexible to deal with emergencies and job changes. In highly specialized and complex working environment such as operating room, operation delay, overtime work or nurse transfers may occur at any time. Traditional scheduling methods are often unable to adapt to these changes quickly, resulting in unreasonable scheduling and affecting work efficiency. The comprehensive collaborative scheduling adopts a dynamic adjustment strategy, which can flexibly adjust the work arrangement of nurses according to the actual situation to ensure the normal operation of the operating room. In addition, comprehensive collaborative scheduling can also improve the job satisfaction and work quality of nurses. The results of this study support this conclusion, and excessive heavy workload often leads to increased fatigue and psychological stress of nurses, which in turn affects their work performance and quality. Comprehensive collaborative scheduling has significant advantages in improving the work efficiency of nurses in the operating room. However, further research is still needed to explore its applicability and effect in different medical institutions and different actual situations.

### 4.2. Influence of comprehensive collaborative scheduling on patient, doctor and nursing satisfaction

Compared with previous studies, this study has made new progress in exploring the effect of operating room nurse scheduling on patient satisfaction. Previous studies mainly focused on the effect of nurses’ scheduling on work efficiency and workload, while there were few studies on patient satisfaction.^[11,12]^ This study found that integrated collaborative scheduling can improve patient, physician and nursing satisfaction, which is a very important finding. Comprehensive collaborative scheduling can ensure that operating room nurses are more attentive and meticulous in the process of work, so as to improve the quality and safety of surgery. Reasonable scheduling can enable nurses to arrive on time, maintain a good working state, and avoid the impact of fatigue and psychological pressure on the quality of work. These factors help to improve the success rate of surgery and reduce the risk of surgery, thereby improving patient satisfaction. Comprehensive collaborative scheduling can also promote communication between nurses and patients, and increase the sense of security and trust of patients during the operation. In the operating room, nurses are the patient closest partner and supporter, and they need to give the patient comprehensive care and concern during the procedure. Through comprehensive collaborative scheduling, nurses can better understand the needs of each patient, and have enough time and energy to provide better services for patients, so as to improve patients’ satisfaction with the work in the operating room. This study also confirmed that comprehensive collaborative scheduling can also improve the evaluation and recognition of nurses by doctors, thereby promoting collaboration and cooperation between doctors and nurses. In the operating room, doctors and nurses are closely working partners who need to cooperate and support each other to accomplish difficult tasks. Through comprehensive collaborative scheduling, nurses can better play their professional skills and experience, and then get the recognition and respect of doctors, so as to improve the satisfaction of doctors and nurses.

### 4.3. Limitations

However, it should be noted that the results of this study may only apply to the situation of primary hospitals, and there may be differences in large medical institutions or specific specialty areas. Primary hospitals usually have small operating rooms and relatively simple types of surgery, so comprehensive collaborative scheduling may be easier to implement and manage. However, in large medical institutions or specific specialty areas, more complex factors and needs may need to be considered, such as the coordination between different surgical departments and the high degree of specialization of specialized surgery, so the effect of comprehensive collaborative scheduling may be different in these cases. In addition, limitations of our study include the small number of patients enrolled and the single-center study design. Due to the small number of patients enrolled, there may be a problem of insufficient sample size, which limits the reliability and generalizability of the study results. Also, the study had a single-center design in which data were collected and analyzed in only one healthcare institution, which may have affected the generalizability of our findings due to facility-specific factors such as the environment, culture, and practices at that institution. To more fully assess the impact of integrated collaborative scheduling on patient satisfaction, future studies could consider increasing the sample size, adopting a multicenter study design, and covering different types of healthcare facilities and specialty areas. In addition, the specific implementation methods, management strategies, and interactions with other factors (such as type of surgery, medical resources, etc) can be further explored to obtain more accurate and comprehensive conclusions.

### 4.4. Significance and suggestions for popularization and application

In this study, comprehensive collaborative scheduling was applied to nursing human resource management in operating room, and it was promoted in a certain range of regions. The results show that this method has important promotion significance, and can provide useful reference for primary hospitals to improve the working efficiency and patient satisfaction of the operating room. Therefore, in the follow-up work, the promotion and popularization of this method can be further strengthened to ensure its function in a wider range of scenarios.

## 5. Conclusions

Comprehensive collaborative scheduling is an effective method of nursing human resource management in operating room, which can effectively improve the work efficiency of nurses and the satisfaction of patients, doctors and nurses. In practice, this method needs to be continuously explored and refined to adapt to different application scenarios and requirements.

## Author contributions

**Writing – original draft:** Lijun Ding.

**Writing – review & editing:** Lijun Ding.
